# Supplementary material for: Indole-3-acetic acid production is rare among gut bacteria and reflects OFOR-driven amino acid oxidation in acetogens
Source: Gut Microbes. 2026 Jun 21;18(1):2689610. doi: 10.1080/19490976.2026.2689610 (PMC13290082; doi:10.1080/19490976.2026.2689610)
Supplement: Supplementary Material.pdf [file KGMI_A_2689610_SM3202.pdf]

Supplementary Material for:

Indole-3-acetic acid production is rare among gut bacteria and reflects OFOR-driven amino acid oxidation in acetogens

Mary E. DeFeo[1,2], Yuanyuan Liu[2], Zhiwei Zhou[2], Steven K. Higginbottom[1], Dylan Dodd[1,2*]

[1]Department of Microbiology and Immunology, Stanford University School of Medicine, Stanford, CA, USA; [2]Department of Pathology, Stanford University School of Medicine, Stanford, CA, USA.

*Correspondence: ddodd2@stanford.edu

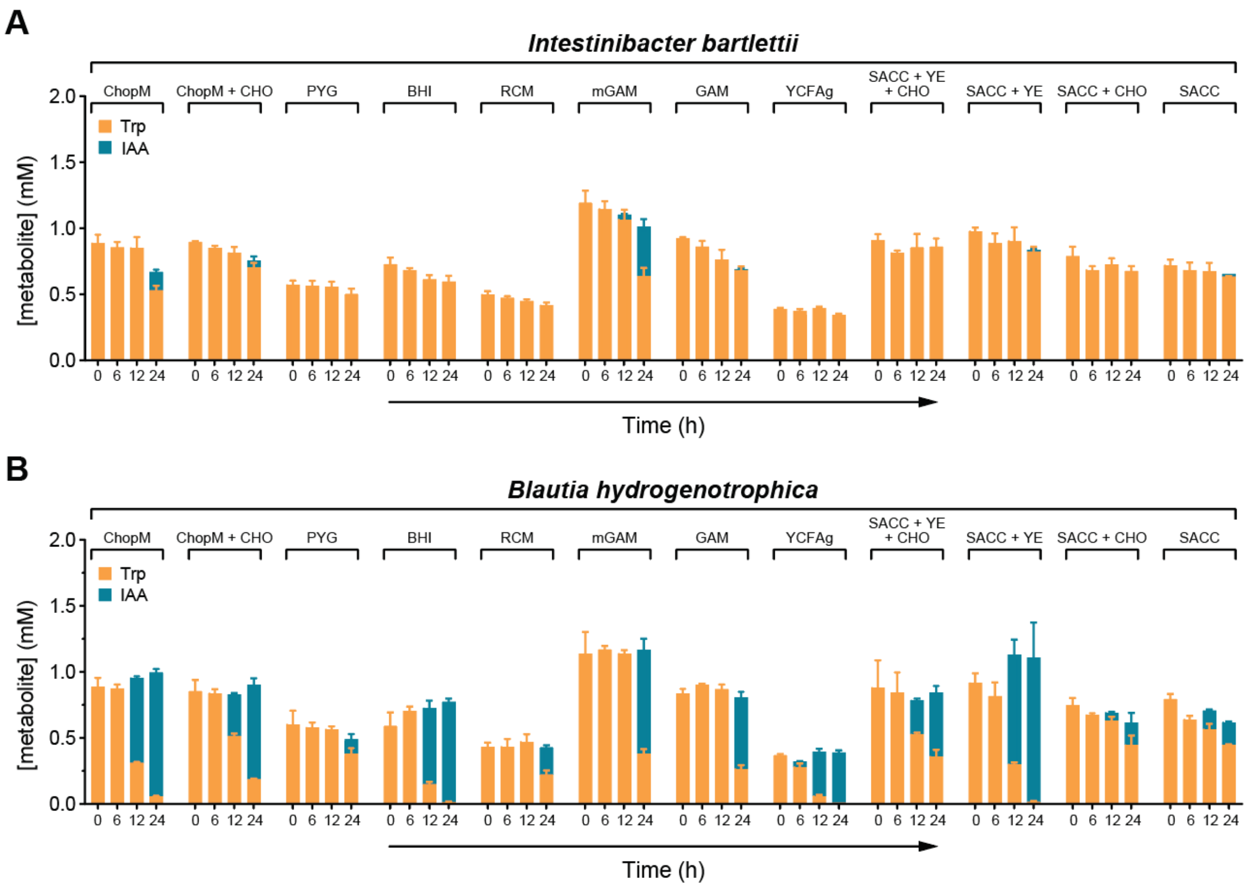

Figure S1. Trp consumption and IAA production in Iba and Bhy cultures in 12 different media. Iba and Bhy were cultured in different media and free Trp and IAA were quantified over time by LC-MS. Data are replotted from Figure 1D and Table S7. Data are plotted as means + SD from n = 3 replicates.

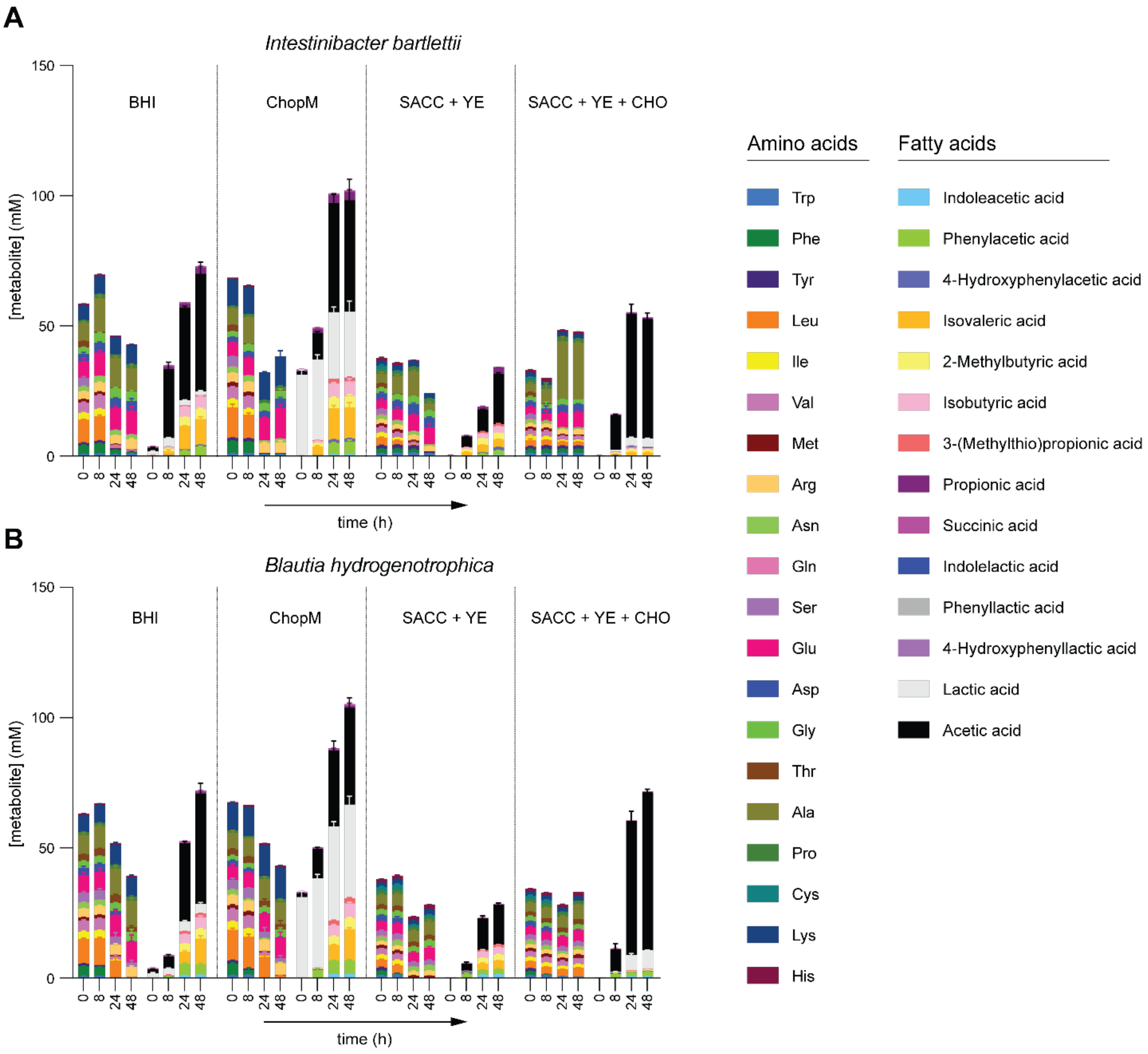

Figure S2. Comprehensive metabolic profiling of *Iba* and *Bhy* in four different media. (A-B) LC-MS time-course profiling of *Iba* (A) and *Bhy* (B) grown in four media, showing amino acid consumption and fatty acid production over time. Data are plotted as means + SD from n = 3 replicates.

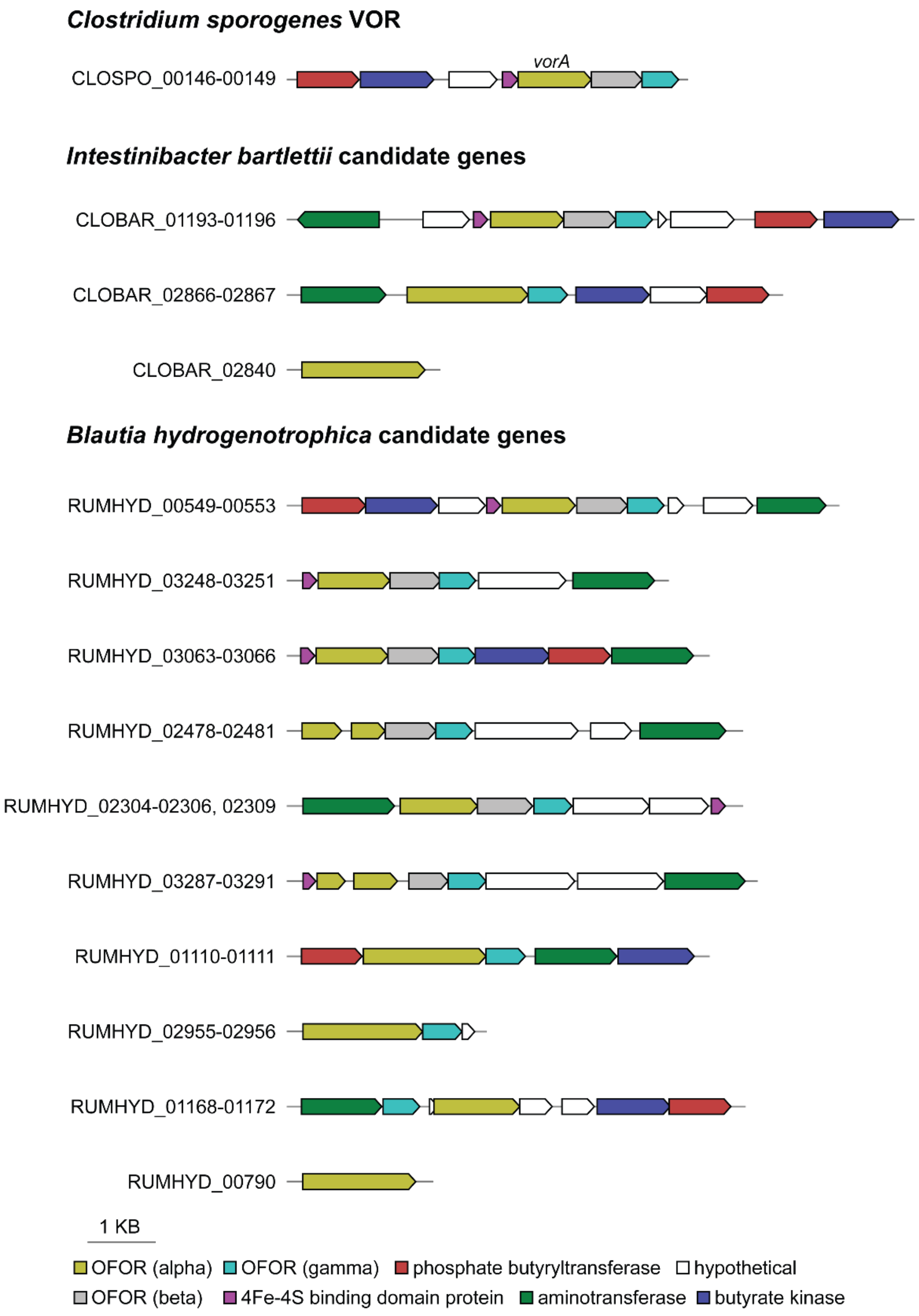

Figure S3. Gene clusters for the known VOR from *C. sporogenes* and candidate OFORs from *Iba* and *Bhy*. The *C. sporogenes* VOR gene cluster with linked genes was used as a query to search the genome sequences of *Iba* and *Bhy* using MultiGeneBlast with an amino acid percent identity cutoff of 15%. The OFOR homologs (CLOBAR_02840 and RUMHYD_00790) were not identified as hits from *C. sporogenes* VOR and they were manually retrieved from the *Iba* and *Bhy* genomes.

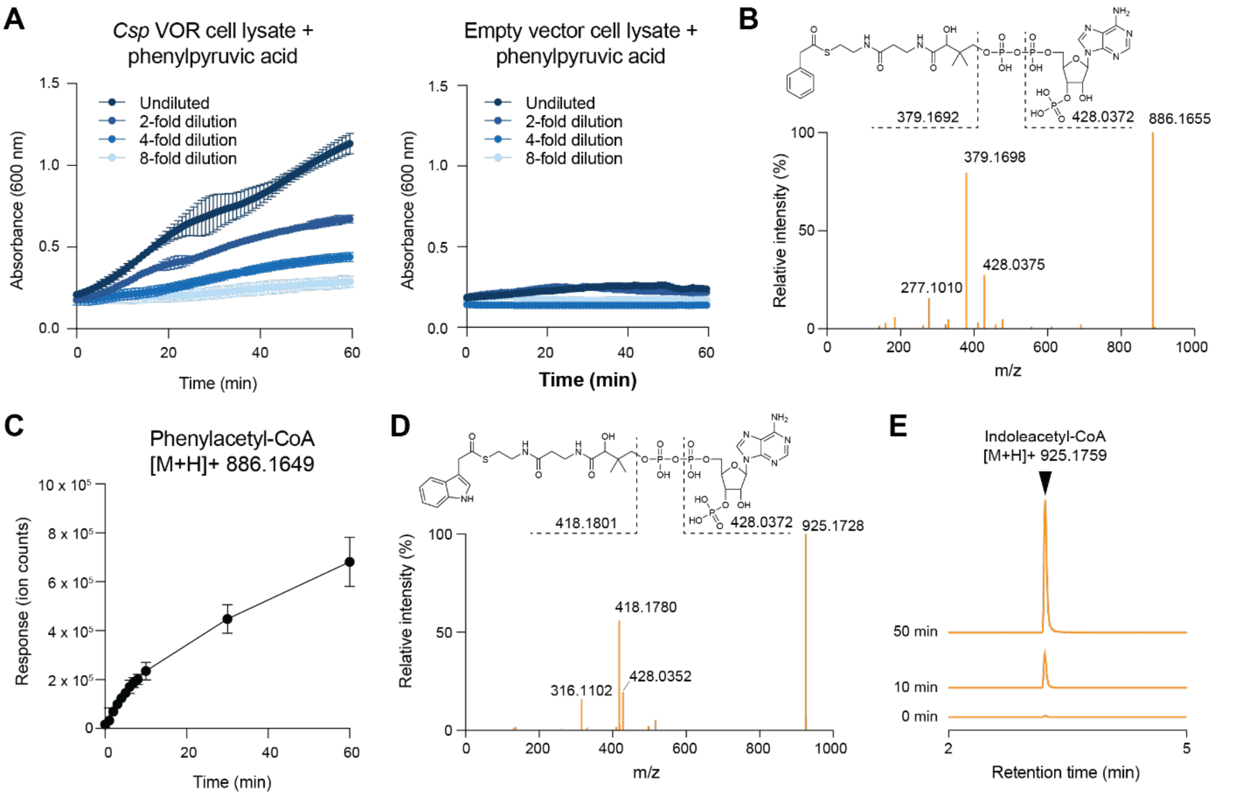

Figure S4. Supporting data for enzyme assays and identification of phenylacetyl-CoA and indoleacetyl-CoA. (A) Methyl viologen reduction progress curves for serial dilutions of *E. coli* cell extracts expressing the *C. sporogenes* VOR or empty vector control, incubated with phenylpyruvic acid. (B) MS/MS spectrum of a candidate phenylacetyl-CoA feature detected in *E. coli* extracts expressing *C. sporogenes* VOR after incubation with phenylpyruvic acid ([M+H]+ 886.1649, RT = 3.09 min). (C) Time-course accumulation of phenylacetyl-CoA in *E. coli* extracts expressing *C. sporogenes* VOR, incubated with phenylpyruvic acid. (D) MS/MS spectrum of a candidate indoleacetyl-CoA feature detected in *E. coli* extracts expressing *Iba* CLOBAR_02866–02867 after incubation with indolepyruvate ([M+H]+ 925.1759, RT = 3.16 min). (E) Extracted ion chromatograms showing an increase in the indoleacetyl-CoA feature ([M+H]+ 925.1759, RT = 3.16 min) over time during incubation of *E. coli* extracts expressing *Iba* CLOBAR_02866–02867 with indolepyruvic acid. For (B) and (D), diagnostic acyl-CoA fragmentation ions are annotated; observed fragment m/z values agree with the predicted acyl-CoA cleavage pattern.

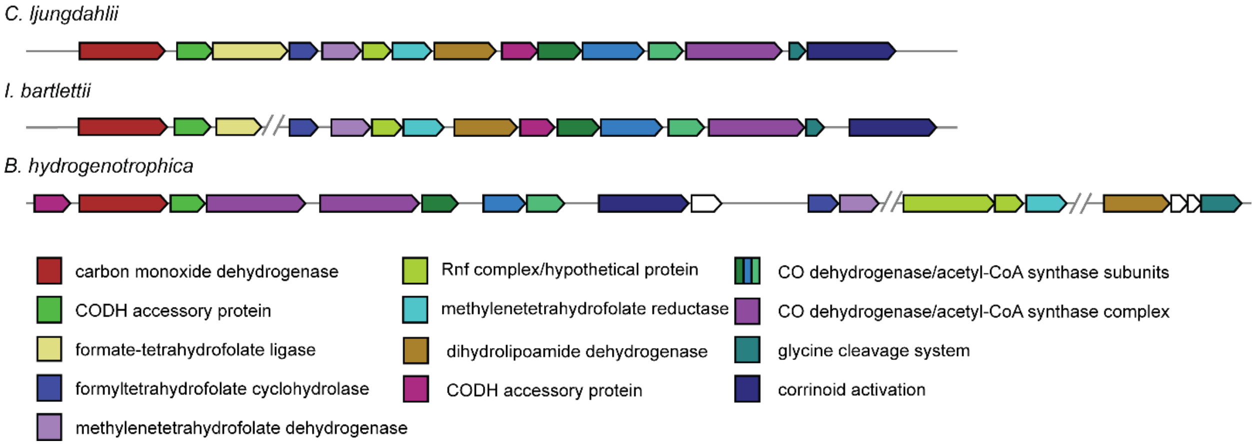

Figure S5. Wood-Ljungdahl Pathway gene clusters in *Iba* and *Bhy*. The *C. ljungdahlii* WLP gene cluster was used as a query to search the genome sequences of *Iba* and *Bhy* using MultiGeneBlast using default parameters.
